# Ultrasound-Guided Percutaneous Tenotomy for Trigger Finger: A Minimally Invasive Office-Based Case

**DOI:** 10.7759/cureus.100487

**Published:** 2025-12-31

**Authors:** Nicholas Lorenz, Amia Mourad, Stephen Westfall, Joe Bhagratie

**Affiliations:** 1 Medical School, Lake Erie College of Osteopathic Medicine, Bradenton, USA; 2 Orthopaedics, Baptist Medical Center Jacksonville, Jacksonville, USA

**Keywords:** a1 pulley, minimally invasive procedure, percutaneous trigger finger release, trigger finger, ultrasound-guided tenotomy

## Abstract

Trigger finger is frequently managed with conservative therapy and corticosteroid injections, while surgery is reserved for refractory cases. Ultrasound-guided percutaneous tenotomy has emerged as a minimally invasive alternative that allows direct visualization of the A1 pulley, flexor tendons, and surrounding neurovascular structures. We present the case of a 70-year-old man with Grade 3 stenosing tenosynovitis of the left middle finger and Grade 2 involvement of the left index finger who experienced only partial improvement after a landmark-based corticosteroid injection. He subsequently underwent ultrasound-guided A1 pulley fenestration with corticosteroid injection in both digits, resulting in complete resolution of pain, triggering, and functional limitation within two weeks. This case demonstrates the safety, efficiency, and clinical effectiveness of ultrasound-guided A1 pulley tenotomy as a quick, office-based intervention for patients who do not achieve full relief from conservative measures.

## Introduction

Trigger finger, aka stenosing flexor tenosynovitis, occurs when repetitive irritation or microtrauma leads to hypertrophy and thickening of the A1 pulley, resulting in narrowing of the fibro-osseous canal through which the flexor digitorum profundus (FDP) and flexor digitorum superficialis (FDS) tendons pass. As the tendon enlarges or the pulley thickens, smooth gliding is impaired, leading to painful clicking, catching, or locking of the digit. This process is analogous to a rope attempting to pass through a rigid ring that has become too tight, resulting in resistance and abrupt motion. When the mismatch between tendon diameter and pulley lumen becomes severe, the digit may become fixed in flexion and require manual extension [1]. This pathophysiologic mismatch explains the hallmark symptoms and the progressive nature of the condition.

Trigger finger affects approximately 2-3% of the general population, with prevalence increasing to up to 20% in individuals with diabetes, making it one of the most common hand disorders encountered in primary care and orthopedics [2]. Standard therapy begins with splinting, NSAIDs, and activity modification, followed by corticosteroid injections, which demonstrate a success rate of up to 85%, particularly in early or mild disease. However, efficacy decreases in cases of multi-digit involvement, prolonged symptom duration, or higher Green classification grades (indicating more severe triggering and locking) [3]. While open surgical A1 pulley release provides >90% long-term success, it requires an incision and postoperative wound care and carries risks such as scar tenderness and neurovascular injury [4]. Blind percutaneous release is less invasive but lacks visualization, increasing risk to the flexor tendons and digital neurovascular bundles [2]. Ultrasound-guided percutaneous tenotomy addresses these limitations by enabling real-time visualization of the pulley, tendon, and neurovascular structures, improving safety and precision while avoiding surgical morbidity [5]. This case highlights the successful use of ultrasound-guided tenotomy in a patient with persistent symptoms despite prior steroid injection.

## Case presentation

A 70-year-old man presented with several months of painful catching and locking of the left index and middle fingers. He rated the pain a 7/10. At times, the middle finger required manual extension to straighten fully. He reported no trauma. He denied associated numbness, paresthesias, or morning stiffness. Physical examination demonstrated palpable tenderness over the A1 pulleys of both digits and reproducible triggering, consistent with Grade 3 stenosing tenosynovitis in the middle finger and Grade 2 involvement in the index finger. Initial treatment consisted of a landmark-guided corticosteroid injection (40 mg triamcinolone with lidocaine), which provided only partial symptomatic improvement, reducing but not eliminating mechanical triggering.

**Figure 1:**
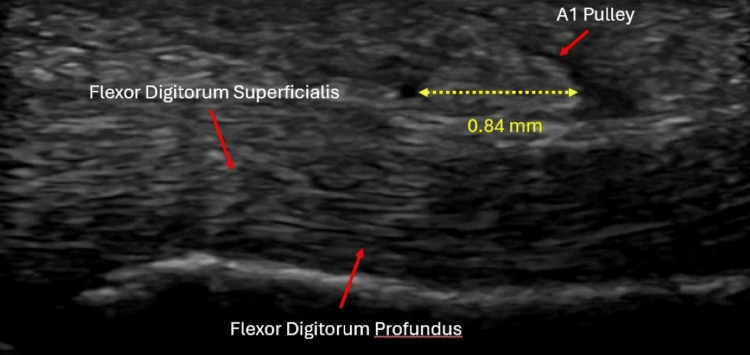
Pre-procedure ultrasound demonstrating A1 pulley thickening (measured at approximately 0.84 mm) and adjacent flexor tendons

**Figure 2:**
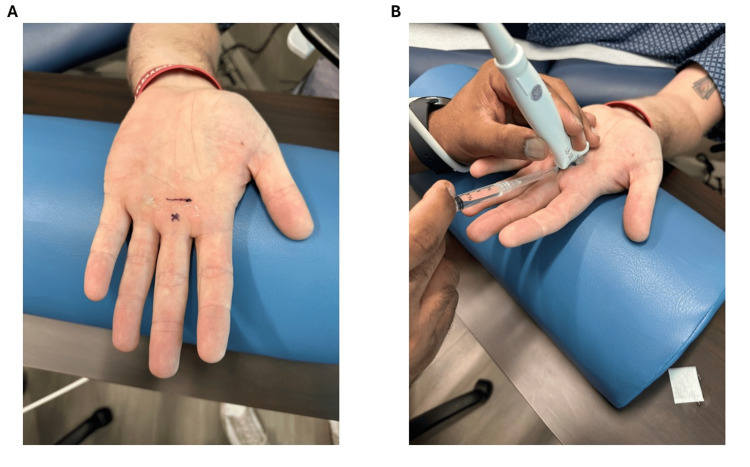
(A) Surface markings identifying the needle insertion point (X) and the level of the A1 pulley (line). (B) Ultrasound-guided needle placement for percutaneous A1 pulley fenestration

Upon evaluation in the Sports Medicine department, ultrasound confirmed A1 pulley thickening in both affected digits (Figure 1). After discussing treatment options, the patient elected to undergo ultrasound-guided percutaneous A1 pulley fenestration. The patient was positioned supine with the hand extended off the edge of the table and stabilized on a padded forearm cushion to allow optimal access and probe control (Figure 2A). Under sterile conditions, the tract was anesthetized with 1% lidocaine using a 27-gauge needle inserted at a 20-30° angle distal to proximal. A 1.5-inch 18-gauge needle was then advanced under long-axis ultrasound guidance, allowing precise visualization of the A1 pulley and underlying flexor tendons (Figure 2B). Approximately 20-40 fenestration passes across the width of the pulley were performed per digit until the pulley tissue softened, and improved tendon excursion was noted sonographically. Through the same needle tract, a mixture of 1% lidocaine and 40 mg triamcinolone was injected.

**Figure 3:**
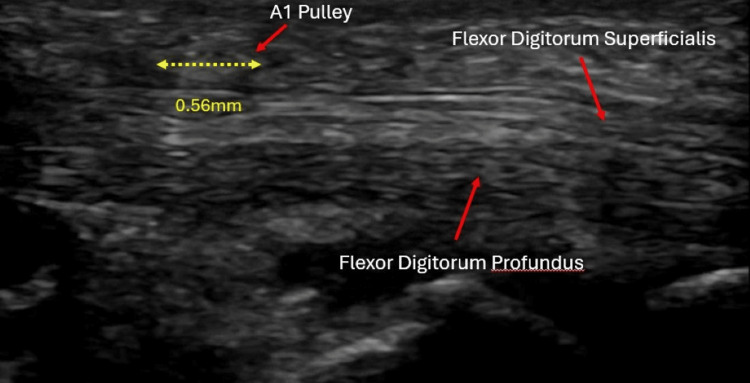
Follow-up ultrasound demonstrating decreased A1 pulley thickening (measured at approximately 0.56 mm) with improved visualization of the flexor tendons.

At his two-week follow-up, the patient reported complete resolution of pain (rating 0/10), catching, and locking, with full return of motion and no complications. Repeat ultrasound demonstrated decreased A1 pulley thickening and smooth tendon gliding (Figure 3). He required no further treatment.

## Discussion

This case demonstrates the clinical effectiveness of ultrasound-guided A1 pulley tenotomy as an intermediate option for patients whose symptoms do not adequately respond to steroid injection. The patient’s initial landmark-based corticosteroid injection yielded only partial improvement, consistent with reported lower success rates in higher-grade and multi-digit disease [3]. The rapid and complete resolution after mechanical fenestration supports the need for structural decompression when the pathophysiologic mismatch between tendon diameter and pulley lumen persists.

Ultrasound-guided tenotomy offers significant advantages over blind percutaneous release and open surgery. Real-time imaging allows the clinician to visualize not only the A1 pulley but also adjacent structures, notably the FDS and FDP tendons and the radial and ulnar digital neurovascular bundles [6]. This visualization reduces risk and increases procedural accuracy. A recent systematic review comparing ultrasound-guided and blind percutaneous trigger finger release reported higher clinical success rates and fewer complications with ultrasound guidance, supporting its role as a safer and more precise minimally invasive alternative to blind techniques [7].

Several structures are at risk during a blind percutaneous release. Flexor tendon injury, including partial-thickness laceration of the FDS or FDP, can lead to pain, weakness, triggering, or delayed rupture. Radial and ulnar digital nerves, which run in close proximity to the A1 pulley, may be injured, resulting in neuropraxia, dysesthesia, or neuroma formation. Digital arteries may be damaged, causing hematoma or compromised distal perfusion [8]. Additionally, inadvertent release of adjacent pulleys such as A2 or A3 can cause bowstringing, altering tendon biomechanics, and impairing grip function. Injury to the tendon sheath or volar plate may also provoke synovitis or joint irritation [9]. Ultrasound guidance greatly mitigates these risks by allowing the operator to confirm needle position and visualize each fenestration pass relative to sensitive structures [10]. 

A limitation in this case is the potential contribution of corticosteroids injected during the procedure. However, the patient’s minimal response to the initial steroid injection suggests that the steroid alone was insufficient for symptom resolution. The immediate improvement in tendon gliding seen on ultrasound following fenestration supports the conclusion that mechanical decompression of the A1 pulley played the primary therapeutic role.

## Conclusions

Ultrasound-guided percutaneous A1 pulley tenotomy is a highly effective, safe, and minimally invasive treatment for trigger finger and is particularly valuable for patients with higher-grade disease or suboptimal response to corticosteroid injection. By providing real-time visualization of the pulley, flexor tendons, and neurovascular structures, this technique minimizes the risk of iatrogenic injury while allowing rapid symptom resolution and functional recovery. This case demonstrates complete restoration of function in two affected digits following a brief, office-based procedure, supporting ultrasound-guided tenotomy as a strong intermediate option between conservative management and open surgical release. While trigger finger may recur and longer-term outcomes cannot be inferred from this limited follow-up, the observed clinical and sonographic improvement suggests a meaningful therapeutic effect. The contribution of the concomitant corticosteroid injection cannot be excluded, and future studies with longer follow-up are needed to improve durability and isolate treatment effects.
